# Hospitalisations related to benzodiazepine, Z-drug, and opioid treatment in Italy: a claim on the risks associated with inappropriate use

**DOI:** 10.1007/s00228-022-03354-7

**Published:** 2022-06-22

**Authors:** Irene Mattioli, Alessandra Bettiol, Giada Crescioli, Roberto Bonaiuti, Domenico Prisco, Guido Mannaioni, Niccolò Lombardi, Alfredo Vannacci, the MEREAFaPS Study group

**Affiliations:** 1 Department of Experimental and Clinical Medicine, University of Florence, Florence, Italy; 2 Section of Pharmacology and Toxicology, Department of Neurosciences, Psychology, Drug Research and Child Health, University of Florence, Viale G. Pieraccini, 6, 50139 Florence, Italy; 3 Tuscan Regional Centre of Pharmacovigilance, Florence, Italy; 4 Medical Toxicology Unit and Poison Control Centre, Careggi University Hospital, Florence, Italy

**Keywords:** Benzodiazepines, Z-drugs, Opioids, Adverse drug reactions, Drug safety, Pharmacovigilance

## Abstract

**Purpose:**

Benzodiazepines (BZD), Z-drugs (ZD), and opioids share a high risk of abuse. This study assessed and characterised adverse events (AEs) related to BDZ, ZD, and opioids leading to emergency department (ED) visits in the Italian setting.

**Methods:**

ED accesses related to BDZ, ZD, and/or opioids were analysed from the MEREAFaPS database. Information on AEs, suspected and concomitant medications was retrieved. Multivariate logistic regression was used to estimate the reporting odds ratios (RORs) of hospitalisation according to the different treatments.

**Results:**

A total of 5,970 pharmacovigilance reports involving BZD/ZD (*n* = 3,106), opioids (*n* = 2,767), or their combination (*n* = 97) were analysed. Compared to opioids, patients with BZD/ZD-related AEs were often younger (51 vs 64 years), more frequently presented 2+ suspected medications (13 vs 3%), and often had a history of abuse (4%). Twenty-three percent of BZD/ZD-related AEs were related to drug abuse (vs 2% of opioid-related ones) and frequently required patient hospitalisation (52% vs 24%), despite the significantly lower clinical complexity of these patients as compared to those on opioids. An increased risk of hospitalisation was found for flurazepam (ROR 1.62; 95% CI, 1.18–2.22), prazepam (2.66; 1.05–6.70), lorazepam (1.26; 1.07–1.49), and morphine (1.76; 1.11–2.79).

**Conclusions:**

These results indicate that, in Italy, the inappropriate use of BZD/ZD is a relevant heath issue, often leading to serious AEs requiring patients’ ED visits and hospitalisation, especially in young women and patients with a history of substance abuse.

**Supplementary information:**

The online version contains supplementary material available at 10.1007/s00228-022-03354-7.

## Introduction

Drug abuse in the world has been a growing problem. In the past two decades, the USA has seen a surge in opioid use and abuse that has led to an increase in the number of overdose deaths estimated at 42 thousand individuals in 2016 [1]. Notably, there has been an exponential increase in fentanyl use over the past 20 years, followed by oxycodone and morphine [2]. Furthermore, considering the widespread use of opioids for pain therapy resulting from conditions such as trauma, surgery, and cancer [3], there has been an increasing need to address this issue to safeguard the right to safe access to these therapies [4].

To date, guidelines discourage the initiation of therapy with long-acting opioids and their use at high doses, and criteria for prescribing opioids suggest remaining on dosages lower than 50 mg/day of oral morphine equivalents (OME), and in any case not exceeding 90 mg/day. In fact, the risk of mortality appears to increase consistently above 40 mg/die of OME, and is even greater when benzodiazepines (BZDs) are used in combination [5].

Abuse of BZD or Z-drugs (ZD) is another growing problem in high-income countries. Between 2.3 and 18% of Americans report to have abused sedatives or tranquillisers for nonmedical use during their lifetime [6].

Consequently, concomitant with the increase in the prescription of these drugs and their abuse, an increase in the number of emergency department (ED) visits for adverse events (AEs) has been observed in both the USA [7, 8] and Europe [9, 10]. As for Italy, according to the 2018 report of the Italian National Observatory on Medicines Use (*Osservatorio Nazionale sull'Impiego dei Medicinali*, OsMed), BZD and ZD are the most purchased medications belonging to the class C category of drug prescriptions, i.e. medications fully paid by the citizen (representing 18.5% of expenditure and 26% of DDD/1000 inhabitants die for the year 2018) [11].

While BZD and ZD are far more used than opioids in Italy, in the period between 2013 and 2018, there has been an increase in the prescription of drugs for pain therapy (from 6.7 DDD/1000 inhabitants die in 2013 to 7.3 in 2018: +9.2%) and an increase in the prevalence of use of 12% (by 5.2% between 2019 and 2018, or 7.7 DDD). In particular, tramadol and fentanyl are the active substances with the highest average DDD/1000 inhabitants per day, 0.8 and 0.6, respectively [11].

Moreover, considering data of ED visits collected in Italy through the MEREAFaPS study between 2007 and 2018, it is possible to observe that the frequency of hospitalisation is always much higher for BZD and ZD than for all other classes of medications that can induce tolerance and dependence, such as opioids [10, 12–16].

In light of this, this post hoc analysis aimed to assess and characterise AEs related to the use of BDZ, ZD, and opioids that led to ED visits and/or hospitalisations in the Italian setting.

## Methods

### Study design and setting

A retrospective study was conducted on the MEREAFaPS database, an Italian database of pharmacovigilance report forms of suspected AEs requiring ED visits, actively collected between January 1st, 2007, and December 31st, 2018, in ED [10, 12–16]. The study involved more than 90 EDs from general hospitals, representative of five Italian Regions (Lombardy, Piedmont, Tuscany, Emilia-Romagna, and Campania). The coordinating centre of Tuscany Region (Italy) approved MEREAFaPS Study (Notification number 1225—December 21, 2009), and the local institutional ethics committee approved MEREAFaPS Study (Study number 3055/2010, Protocol number 45288—August 6, 2014) according to the legal requirements concerning observational studies.

### Exposures

Only pharmacovigilance reports with BZD (ATC classes: N05BA*, N05CD*), ZD (N05CF*), and/or opioids (N02AA*, N02AB*, N02AE*, N02AG*, N02AJ*, N02AX*) as suspected medications were considered [17]. Patients who developed an AE while in the ED for any other reason rather than BZD, ZD, or opioids were excluded.

Suspected medications were classified in terms of pharmacological class and active substance. BZD and ZD were classified based on their plasma half-life as short-, intermediate-, or long-acting [10, 18, 19], while opioids were classified as weak or strong [10, 18–20], based on current pharmacology literature. Moreover, information on concomitant medications was also considered.

When available, information on drug misuse, abuse, medication error, or overdose, defined according to the Good Pharmacovigilance Practices (GVP)—Module VI (Rev 2) [21], was also collected. In particular, a misuse refers to an intentional and inappropriate use of a medication not in accordance with the information reported in the SPC. An abuse is defined as a persistent or sporadic, intentional excessive use of a medicinal product, accompanied by harmful physical or psychological effects. On the contrary, a medication error refers to any unintentional error in the prescribing, dispensing, or administration of a medicinal product under the control of a healthcare professional. Finally, an overdose refers to the administration of a quantity of a medicinal product which is above the maximum recommended dose according to the authorised product information.

### Outcomes

AEs were coded using the Medical Dictionary for Regulatory Activities (MedDRA) and reported as System Organ Class (SOC) and Preferred Term (PT) [22]. The total number of times each PT was recorded within all pharmacovigilance report forms was calculated. AEs requiring patient’s hospitalisation were considered serious. All patients admitted to hospital wards or those referred to intensive short observation units were considered hospitalised. Moreover, AE outcomes were assessed and classified as resolution with sequelae, still unresolved, complete resolution, improvement, death, and not available.

### Statistical analysis

Descriptive statistics were used to summarise data. Categorical data were reported as frequencies and percentages and compared using the chi-square test, while continuous data were reported as median values with the related interquartile ranges (IQR) and compared using the Mann–Whitney test for unpaired data.

Within the BZD/ZD group, univariate logistic regression models were fitted to estimate the reporting odds ratios (RORs) of hospitalisation with 95% confidence intervals (CIs) associated with short-, intermediate-, and long-acting BZD or with ZD, respectively, as compared to the other four pharmacological classes. Moreover, RORs of hospitalisation were estimated for each considered active substance, each one compared to all other active substances belonging to BZD/ZD.

Similarly, for the opioids group, univariate logistic regression models were fitted to estimate the RORs of hospitalisation with 95% CIs associated with strong, weak opioids or fixed associations containing opioids, respectively, each one compared to the other two pharmacological classes.

Multivariate logistic regression models were also computed, adjusting for age, sex, ethnicity, presence of concomitant medications, and presence of concomitant conditions.

All results were considered to be statistically significant at *p* < *0.05*. Data management and statistical analysis were performed using the software STATA 17 (StataCorp, USA).

## Results

During the study period (2007–2018), 5,970 AE reports involving BZD or ZD (*n* = 3,106, BZD/ZD group), opioids (*n* = 2,767, OP group), or their combination (*n* = 97, BZD/ZD + OP group) were collected (Table 1). Patients experiencing AEs related to opioids were significantly older as compared to those in the other two groups, the median age being 63.8 (47.1–77.7) years in the OP group, 51.3 (39.1–70.7) years in BZD/ZD group, and 58.8 (45.5–79.9) years in the BZD/ZD + OP group (overall *p* = *0.0001*). Overall, female sex was significantly more represented, particularly in the OP group. In most cases, only one suspected medication was involved, mostly administrated by oral route. The presence of two or more suspected medications was significantly more frequent in reports related to BZD/ZD (12.7% in the BZD/ZD group vs 3.1% in the OP group); in the BZD/ZD + OP group (where at least two suspected medications were involved by definition), the co-presence of a third (or more) agent was reported in 22.7%. Of major note, in around one-fourth of AE reports related to BZD/ZD or to BZD/ZD + OP, a drug abuse or misuse was reported (23.2% and 24.7%, respectively); this percentage was significantly lower for the OP group (1.9%, overall *p* < *0.0001*). Also overdose and therapeutic errors were more frequently reported in the BZD/ZD group, though their frequency was overall low (2.4% and 1.3%, respectively; ≤ 1% for both overdose and therapeutic errors in the other groups). Notably, more than half of AEs related to BZD/ZD or to BZD/ZD + OP required patient’s hospitalisation (52.1% and 51.6%, respectively); this proportion was significantly lower in the OP group (24.4%, *p* < *0.0001*). In all groups AEs usually ended with an improvement or with a complete resolution of symptoms. Of particular interest, four cases of “death” have been observed in the BZD/ZD group and one case in the OP group. In the first group, deceased patients were all females aged ≥ 75 years (mean age 84 years), for whom the causality assessment was consistent with a “possible” relationship between the events and the suspected drugs (i.e. zolpidem, delorazepam, triazolam, and diazepam). In all cases an overdose or abuse of BZD/ZD was reported, and two of them also presented a psychiatric comorbidity. In the second group, the patient who died was a 55-year-old woman of unspecified ethnicity, treated with morphine and oxycodone for the management of cancer pain. No abuse/medication error/overdose was reported.

**Table 1:** Study population

**Reported AEs**	**BZD/ZD group**	**OP group**	**BZD/ZD + OP group**	***p*-value**
	***N* = 3106 (%)**	***N* = 2767 (%)**	***N* = 97 (%)**	
**Age, year**				
≤5	31 (1.0)	3 (0.1)	0 (0)	<0.0001*
6–19	116 (3.7)	33 (1.2)	0 (0)	
20–64	1988 (64.0)	1385 (50.1)	54 (55.7)	
65–79	513 (16.5)	787 (28.4)	20 (20.6)	
≥80	424 (13.7)	539 (19.5)	22 (22.7)	
*Not available*	34 (1.1)	20 (0.7)	1 (1.0)	
Median age (IQR), years	51.3 (39.1–70.7)	63.8 (47.1–77.7)	58.8 (45.5–79.9)	0.0001*
**Sex**				
Female	2080 (67.0)	1952 (70.6)	70 (72.2)	0.010*
Male	1026 (33.0)	815 (29.5)	27 (27.8)	
**Ethnic group**				
Asiatic	29 (0.9)	47 (1.7)	0 (0)	0.001*
Black/Afro- American	14 (0.5)	34 (1.2)	1 (1.1)	
Caucasian	2717 (87.5)	2343 (84.7)	81 (83.5)	
Other	346 (11.1)	343 (12.4)	15 (15.5)	
**Number of suspected medications**				
1	2646 (85.2)	2666 (96.4)	0 (0)	<0.0001*
2	395 (12.7)	87 (3.1)	75 (77.3)	
3+	65 (2.1)	14 (0.5)	22 (22.7)	
**Administration route**				
Total	*N* = 3642	*N* = 2884	*N* = 224	
Enteral	3593 (98.6)	2537 (88)	204 (91.1)	<0.0001*
Parenteral	49 (1.4)	132 (4.6)	12 (5.4)	
Transdermic	0 (0)	194 (6.7)	8 (3.5)	
*Not available*	0 (0)	21 (0.7)	0 (0)	
**AEs due to**				
Abuse/misuse	721 (23.2)	53 (1.9)	24 (24.7)	<0.0001*
Overdose	74 (2.4)	18 (0.7)	1 (1.0)	<0.0001*
Therapeutic error	40 (1.3)	21 (0.8)	0 (0)	0.079
**Hospitalisation**				
No	1489 (47.9)	2093 (75.6)	47 (48.5)	<0.0001*
Yes	1617 (52.1)	674 (24.4)	50 (51.6)	
**Outcome**				
Complete resolution	1234 (39.7)	970 (35.1)	27 (27.8)	<0.0001*
Improved	1412 (45.5)	1438 (52.0)	47 (48.5)	
Still unresolved	86 (2.8)	75 (2.7)	7 (7.2)	
Resolution with sequelae	17 (0.6)	0 (0)	0 (0)	
Death	4 (0.1)	1 (0.0)	0 (0)	
*Not available*	353 (11.4)	283 (10.2)	16 (16.5)	

*AE* adverse event, *BZD* benzodiazepine, *IQR* interquartile range, *OP* opioid, *ZD* Z-drug

*All results were considered to be statistically significant at p < 0.05

The most frequently reported AEs for the BZD/ZD, OP or BZD/ZD + OP groups are reported in Table 2. In the 3,106 AE reports related to BZD/ZD, 6,804 distinct AEs were reported as PT, with 3,680 (54.1%) of them requiring patients’ hospitalisation. The most frequent AEs were related to drowsiness (20.7%), drug abuse or overuse (18.2%), or to an altered mental status (4.7%). Notably, self-harm and attempt to commit suicide accounted for 2.8% and 2.2% of AEs, respectively.

**Table 2:** Most frequently reported adverse events as preferred terms

Preferred term*	**N. reported AEs** **(% in columns)**	**N. AEs requiring hospitalisation** **(% in columns)**
**BZD/ZD group**	***N* = 6804**	***N* = 3680**
Drowsiness	1408 (20.7)	860 (23.4)
Abuse/overuse	1238 (18.2)	780 (21.2)
Altered mental status	317 (4.7)	154 (4.2)
Presyncope or syncope	219 (3.2)	123 (3.3)
Bradyphrenia	195 (2.9)	128 (3.5)
Self-harm	190 (2.8)	134 (3.6)
Toxicity (not specified)	175 (2.6)	86 (2.3)
Attempt to commit suicide	147 (2.2)	106 (2.9)
Bradykinesia	139 (2.0)	96 (2.6)
Asthenia	128 (1.9)	42 (1.1)
**OP group**	***N* = 6449**	***N* = 1636**
Nausea and vomiting	1525 (23.6)	313 (19.1)
Abdominal pain/constipation	566 (8.8)	150 (9.2)
Dizziness	494 (7.7)	71 (4.3)
Malaise	307 (4.8)	50 (3.1)
Presyncope or syncope	283 (4.4)	66 (4.0)
Asthenia	214 (3.3)	37 (2.3)
Drowsiness	195 (3.0)	89 (5.4)
Altered mental status	194 (3.0)	67 (4.1)
Hyperhidrosis	164 (2.5)	36 (2.2)
Headache	114 (1.8)	22 (1.3)
**BZD/ZD + OP group**	***N* = 252**	***N* = 147**
Drowsiness	48 (19.0)	32 (21.8)
Drug abuse	25 (9.9)	16 (10.9)
Altered mental status	21 (8.3)	12 (8.2)
Nausea and vomiting	12 (4.8)	3 (2.0)
Vertigo	8 (3.2)	1 (0.7)
Self-harm	7 (2.8)	6 (4.1)
Coma	7 (2.8)	6 (4.1)
Hypotension	7 (2.8)	6 (4.1)
Bradyphrenia	5 (2.0)	3 (2.0)
Attempt to commit suicide	4 (1.6)	4 (2.7)

*AE* adverse event, *BZD* benzodiazepine, *OP* opioid, *ZD* Z-drug

^*^The table shows the number of times that each PT was reported within all pharmacovigilance report forms

In the 2,767 AE reports related to OP, 6,449 distinct AEs were reported as PT, and 1,636 (25.4%) of these events led to hospitalisation. Most AEs were retrievable to gastrointestinal symptoms (nausea and vomiting in 23.6%, abdominal pain and constipation in 8.8%), to neurological (including dizziness, presyncope and syncope, altered mental status, headache), or to generalised symptoms (including malaise, asthenia, and drowsiness).

As for the BZD/ZD + OP group, 252 AEs reported as PT were detectable from the 97 AE reports. A not negligible proportion of AEs were related to drug abuse (9.9%), self-harm (2.8%), or to attempted suicide (1.6%).

Information on concomitant (non-suspected) medications and concomitant conditions is available in Supplementary Table 1. In the OP group, one or more concomitant medications were reported in 52% of cases, and mainly included cardiovascular or gastroprotective medications. Conversely, in the BZD/ZD and in the BZD/ZD + OP groups, concomitant medications were significantly less reported (42% and 49%, respectively), and frequently included drugs targeting the central nervous system (i.e. antidepressants, antipsychotics, antiepileptics). When considering concomitant conditions (which were reported in around 40–50% of cases), anxiety, depressive, and psychiatric disorders were among the most frequently reported conditions in the BZD/ZD and in the BZD/ZD + OP groups. In the OP group, instead, musculoskeletal painful conditions appeared as one of the most frequent underlying conditions.

Table 3 and Supplementary Table 2 summarise the estimated risks of hospitalisation for BZD/ZD. No significant difference emerged between these four pharmacological classes in terms of related risk of hospitalisation. When considering each active substance separately, the two long-acting BZDs flurazepam (adjusted ROR 1.62; CI 95%, 1.18–2.22) and prazepam (adjusted ROR 2.66; CI 95%, 1.05–6.70), and the intermediate BZD lorazepam (adjusted ROR 1.26; CI 95%, 1.07–1.49) were all associated with an increased risk of hospitalisation as compared to the other active substances belonging to the BZD or ZD classes. Conversely, midazolam (a short-acting BZD) was associated with a significantly lower risk of hospitalisation (adjusted ROR 0.21; CI 95%, 0.07–0.64).

**Table 3:** Risk of hospitalisation associated with benzodiazepines/Z-drugs and opioids

**Suspected medications**	**Suspected AEs**	**Suspected AEs requiring hospitalisations**	**Crude ROR**	Adjusted ROR*
**BZD/ZD group**				
	***N* = 3642 (%)**	***N* = 1959 (%)**		
Long-acting BZD	915	512	1.12 (0.97–1.31)	1.05 (0.90–1.23)
Intermediate-acting BZD	2087	1124	1.01 (0.88–1.15)	1.04 (0.91–1.19)
Short-acting BZD	282	144	0.89 (0.70–1.13)	0.90 (0.70–1.15)
Z-drugs	358	179	0.84 (0.68–1.05)	0.87 (0.70–1.08)
**OP group**				
	***N* = 2884 (%)**	***N* = 727 (%)**		
Strong opioids	574	197	1.75 (1.44–2.14)	1.53 (1.25–1.87)
Minor opioids	711	153	0.76 (0.62–0.94)	0.83 (0.68–1.02)
Fixed associations	1599	377	0.82 (0.70–0.98)	0.85 (0.72–1.01)

*AE* adverse event, *BZD* benzodiazepine, *OP* opioid, *ROR* reporting odds ratio, *ZD* Z-drug

^*^Adjusted by age, sex, Caucasian ethnicity, presence of concomitant drugs and concomitant conditions

Table 3 and Supplementary Table 3 summarise the estimated risks of hospitalisation for opioids. Strong opioids resulted to be associated with a significantly enhanced risk of hospitalisation as compared to weak opioids or fixed associations (adjusted ROR 1.53; CI 95%, 1.25–1.87). Namely, when considering the different active substances, morphine resulted to be the opioid associated with the highest risk of AE-related hospitalisation (adjusted ROR 1.76; CI 95%, 1.11–2.79), while the fixed association of tramadol/dexketoprofen was associated with the lower risk (adjusted ROR 0.26; CI 95%, 0.13–0.54).

## Discussion

This study aimed to describe the frequency and pharmacological characteristics of BZD, ZD, and opioid-related AEs leading to ED visits and/or hospitalisations.

In terms of age, subjects who experienced AEs from BZD and/or ZD were significantly younger than those who experienced AEs from opioids with a median age of 51.3 (39.1–70.7) and 63.8 (47.1–77.7) years, respectively. This finding allows us to frame the latter as older subjects. As for gender, the female sex was the prevalent one, especially among subjects experiencing opioid-related AEs. These results are comparable with those published by Bushnell and colleagues in their study characterising BZD poisoning ED visits in young people by sex [23]. In particular, authors found that the high proportion of BZD-related ED visits were intentional and included mental health disorder diagnoses (i.e. depression), especially among young females.

When considering concomitant conditions, results underlined a more severe clinical complexity for subjects in the OP group, which resulted to be frequently affected by hypertension and diabetes. Conversely, in the BZD/ZD group we found anxiety, depressive, and psychiatric disorders (i.e. sleep disorder) among the most frequently reported concomitant conditions, which generally represent some of the therapeutic indications of these medications. Also, history of substance abuse was reported in around 4% of patients in the BZD/ZD group. It is worth mentioning that the presence of substance abuse as a common concomitant condition could indicate poor appropriateness, both in the prescription and use of BZD/ZD, as the use of BZD/ZD is generally contraindicated among these subjects [24]. Our evidence can be compared with the results by Kurtz and colleagues, who reported in their multivariate logistic regression model that younger age, severe mental distress, daily marijuana use, and heavy opioid use are associated with BZD misuse/abuse and dependence [25].

Regarding concomitant medications, we found that, in the BZD/ZD group, antidepressants and antipsychotics were frequently co-administered, due to the common therapeutic practice of associating BZD to antidepressants in the first 2–3 weeks of the treatment of some mood disorders [24]. On the other hand, for the OP group, the most frequently reported concomitant drugs were antithrombotics. Noteworthy, aspirin-like drugs also belong to this class, which, together with antihistamines and antidepressants, may increase the analgesic effects of opioids and, potentially, also increase their toxicity [18]. Despite this higher clinical complexity in the OP group, only 3.6% of subjects who had opioid-related AEs presented two or more suspected medications, as compared to approximately 15% in the BZD/ZD group. This finding could indicate a greater prescriptive appropriateness in the OP group, likely due to their complexity and frailty, suggesting that healthcare professionals (i.e. general practitioner, specialist, pharmacist, etc.) are probably more careful in the management of patients on opioid therapy than those on BZD/ZD treatment.

Particular attention should be paid to the concomitant use of BZD/ZD and opioids. Despite that in our analysis the concomitant use of both drug classes accounted for a small amount of cases and did not show an increase of hospitalisations or poor outcomes of AEs, a study published in 2017 highlighted that, from 2001 to 2013, concurrent BZD/opioids use suddenly increased in the USA and significantly contributed to the overall population risk of opioid overdose [26]. Conversely, BZD misuse poses at risk the health and treatment of high-risk opioid users, people among whom these medications have been implicated in considerable numbers of drug-related deaths, thus fuelling a vicious cycle with clonazepam, diazepam, and alprazolam as the most frequently reported BZDs in opioid abusers [27].

In this context, it is worth remembering that, while opioids are mostly paid by the National Health System, in Italy BZD/ZD are always paid by patients, exposing them to a less strict monitoring by physicians, especially in terms of regular ambulatory follow-up. Coherently, our results indicated that abuse and misuse of BZD/ZD was much more frequent than for opioids, accounting for around 20% of AEs requiring ED visit in the BZD/ZD group, as compared to 2% in the OP group. Similarly, overdose and therapeutic errors were significantly more frequent in the BZD/ZD group (2.4% vs < 1%, respectively). Furthermore, BZDs are the only class of medications reported among a range of drug harms in the UK by Nutt and colleagues through their multicriteria decision analysis performed [28]. In the ranking reported by the authors, BZDs are in 10th position among the substances causing harm, immediately after the most well-known drugs (i.e. alcohol, heroin, cocaine, etc.). Moreover, in Italy BZD/ZD are relatively inexpensive drugs (both as brand and equivalent), in particular those most commonly prescribed (lormetazepam, lorazepam, and alprazolam), having an average retail cost of 6.02 euros/package [11]. This makes them very easy to purchase, exposing individuals who make uncontrolled use of them to a greater risk of abuse/misuse and even serious AEs which often require patients’ hospitalisation.

Of note, we also found that the hospitalisation rate for subjects in the BZD/ZD group was about twofold higher than the rate observed for subjects in the OP group (>50% vs <25% in the OP group). Particularly, within the BZD/ZD group, an increased risk of hospitalisation was found for two long-acting BZDs (flurazepam and prazepam) and for an intermediate-acting BDZ (lorazepam), while the short-term-acting midazolam was associated with a lower risk. As reported in our previous study [10], the association between longer BZD plasma half-life and the risk of negative clinical outcomes (mostly falls, fractures, and accidents) is well described in literature [29]. As for midazolam, the lower risk of hospitalisation associated with this medication is likely to be related to its pharmacological properties, as it has a very short plasma half-life and is available on the Italian market mostly as parenteral formulation, making abuse/misuse more difficult [10]. Indeed, oral formulations of BZD, especially drop formulations, are known to be associated with a greater risk of abuse [30].

Regarding opioids, an increased risk of hospitalisation was found for strong opioids (particularly morphine) as compared to weak opioids or fixed associations (particularly tramadol/dexketoprofen). These results are likely to be mostly driven by the high clinical complexity of patients receiving strong opioids, whose complications need to be mostly managed in an in-hospital setting.

In light of this, is BZD/ZD misuse the next drug epidemic? Healthcare professionals, patients, and customers continue to view BZD/ZD use as less harmful than that of opioids. The National Institute on Drug Abuse (NIH) reported 8,791 overdose deaths in 2015 involving BZD, more than 7,600 overdose deaths since 1999. Such a significant increase in the number of deaths should raise alarm worldwide, not only in the USA [31].

In the near future, clinical pharmacologists and toxicologists should take into account the harmful effects of substance use disorder also associated with BZD and ZD [32]. To minimise the phenomenon of inappropriate use of BZD and ZD in the general population, even community pharmacists operating on the national territory should always guarantee the correct dispensing of these drugs.

### Limitations and strengths

The results from this study suffer some limitations, mostly related to its retrospective and observational nature, which might have led to an underestimation of the number of AEs analysed. Indeed, not all patients with an AE refer to the ED or spontaneously report the event. However, considering that AEs leading to ED visit were collected through an active pharmacovigilance project, the problem of underreporting, particularly for serious AEs, was minimised in this post hoc analysis. In addition, analyses are based on the collection of pharmacovigilance report forms that could be affected by inaccurate or incomplete information, mainly due to the lack of demographic and/or clinical data. In particular, data on dosages of suspected medications were not available in all pharmacovigilance report forms. Thus, the impact of BZD/ZD or OP dosages on the risk of hospitalisation was not evaluated. Considering cases of misuse, abuse, overdose, or medication errors, these covariates were only considered categorical variables when reported in the pharmacovigilance report form.

Despite these limitations, this study is strengthened by the fact that it involved numerous EDs of hospitals equally distributed throughout the National territory, so we can say that the data collected and the evidence described can be considered representative of all Italian EDs. Moreover, since BZD and ZD are not reimbursed by the Italian National Health Service, it is currently very difficult to conduct safety studies based on prescription data of these drugs. In this context, the use of data related to ED visits and/or hospitalisations represent a unique opportunity to estimate the true impact of BZD/ZD abuse and inappropriate use in the Italian population.

## Conclusions

In the ED setting, active pharmacovigilance studies appear to be a valid scientific approach, enabling both healthcare providers and systems to detect and characterise AEs, particularly those associated with inappropriate use of central nervous system medications, such as BZD, ZD, and opioids. This approach is also useful in the monitoring of the safety of medications which are generally paid for by patients. In fact, for such medications the analysis based on administrative data (i.e. administrative database of reimbursed medicines and/or insurance database) could not be performed.

The results from this study indicate that, while in the USA the opioid epidemic is a major health issue, in Italy the inappropriate use of BZD and ZD is a much more relevant challenge, often leading to serious AEs requiring patients’ ED visits and, eventually, hospitalisation, especially in young women.

The results from this study provide interesting hints for prescribers and emergency physicians for the recognition and management of AEs associated with these pharmacological classes. In particular, specific attention should be paid to young BZD and ZD users with a history of substance abuse.

Increasing the awareness of the potential public health consequences of inappropriate use of BZD and ZD can substantially reduce the number and costs associated with AE-related ED visits and/or hospitalisations, as well as improve patients’ quality of life across all age groups.

## Supplementary Information

Below is the link to the electronic supplementary material.

Supplementary file1 (DOCX 25 kb)

## Data Availability

The data that support the findings of this study are available from the corresponding author upon reasonable request.
