# Supplementary material for: Hospitalisations related to benzodiazepine, Z-drug, and opioid treatment in Italy: a claim on the risks associated with inappropriate use
Source: Eur J Clin Pharmacol. 2022 Jun 22;78(9):1511–9. doi: 10.1007/s00228-022-03354-7 (PMC9365734; doi:10.1007/s00228-022-03354-7)
Supplement: Supplementary file 1 — Supplementary file1 (DOCX 25 kb) [file 228_2022_3354_MOESM1_ESM.docx]

**SUPPLEMENTARY MATERIAL**

**Supplementary table 1.** Concomitant non-suspected medications and conditions

|  | **BZD and ZD**  **(BZD/ZD group)** | **Opioids**  **(OP group)** | **Combination of BZD/ZD and opioids**  **(BZD/ZD+OP group)** | ***p-value*** |
| --- | --- | --- | --- | --- |
|  | **N=3106 (%)** | **N=2767 (%)** | **N=97 (%)** |  |
| **Concomitant medications** |  |  |  |  |
| No | 1806 (58.2) | 1328 (48.0) | 50 (51.6) | <0.0001* |
| Yes | 1300 (41.9) | 1439 (52.0) | 47 (48.5) |  |
| *Most frequently reported medications* | *Psychoanaleptics 554 (17.8)* | *Antithrombotic 545 (19.7)* | *Gastroprotectives 22 (22.7)* |  |
|  | *Antithrombotics 390 (12.6)* | *Gastroprotectives 475 (17.2)* | *Antithrombotics 15 (15.5)* |  |
|  | *Antipsychotics 341 (11.0)* | *Beta-blockers 347 (12.5)* | *Antidiabetics 12 (12.4)* |  |
|  | *Gastroprotectives 266 (8.6)* | *Diuretics 326 (11.8)* | *Psychoanaleptics 11 (11.3)* |  |
|  | *Antiepileptics 259 (8.3)* | *ACE-inhibitors 277 (10.0)* | *Antiepileptics 10 (10.3)* |  |
| **Concomitant conditions** |  |  |  |  |
| No | 1704 (54.9) | 1674 (60.5) | 49 (50.5) | <0.0001* |
| Yes | 1402 (45.1) | 1093 (39.5) | 48 (49.5) |  |
| *Most frequently reported conditions* | *Anxiety and depressive disorders 545 (17.5)* | *Hypertension 330 (11.9)* | *Anxiety and depressive disorders 15 (15.5)* |  |
|  | *Psychiatric disorders 237 (7.6)* | *Diabetes 108 (3.9)* | *Hypertension 8 (8.2)* |  |
|  | *Hypertension 217 (7.0)* | *Neck or back pain 93 (3.4)* | *Obesity 5 (5.2)* |  |
|  | *Drug abuse 116 (3.7)* | *Cardiovascular disorders 65 (2.3)* | *Diabetes 4 (4.1)* |  |
|  | *Diabetes 95 (3.1)* | *Discopathy 65 (2.3)* | *Cognitive deficiencies 4 (4.1)* |  |

**Supplementary table 2.** Risk of hospitalization associated with the different active substances belonging to benzodiazepines/Z-drugs

| **Suspected**  **medications** | **Plasmatic half-life, hours** | **Suspected adverse events** | **Suspected adverse events requiring hospitalizations** | **Crude ROR** | **Adjusted ROR*** |
| --- | --- | --- | --- | --- | --- |
|  |  | **N=3642 (%)** | **N=1959 (%**)** |  |  |
| **Benzodiazepines/Z-drugs** | | | | | |
| **Long-acting BZD** | **≥24** | 915 | 512 | 1.12  (0.97-1.31) | 1.05  (0.90-1.23) |
| Delorazepam | 98.3 (80-115) ^b,#^ | 420 (11.5) | 222 (52.9) | 0.96  (0.78-1.18) | 0.90  (0.73-1.11) |
| Flurazepam | 74±24 ^a,°^ | 179 (4.9) | 118 (65.9) | **1.70**  **(1.24-2.34)** | **1.62**  **(1.18-2.22)** |
| Prazepam | 60 (36-200) ^b,#^ | 25 (0.7) | 19 (76.0) | **2.74**  **(1.09-6.87)** | **2.66**  **(1.05-6.70)** |
| Nordazepam | 56 (36-200) ^b,#^ | 1 (0.0) | 1 (100) | - | - |
| Ketazolam | 43 (26-200) ^b,#^ | 1 (0.0) | 1 (100) | - | - |
| Diazepam | 43±13 ^a,°^ | 264 (7.3) | 140 (53.0) | 0.97  (0.75-1.24) | 0.93  (0.72-1.19) |
| Clobazam | 36-42 ^b,^^ | 22 (0.6) | 9 (40.9) | 0.59  (0.25-1.39) | 0.52  (0.22-1.23) |
| Nitrazepam | 26 (15-38) ^b,#^ | 3 (0.1) | 2 (66.7) | 1.72  (0.16-8.97) | 1.81  (0.16-0.25) |
| **Intermediate-acting BZD** | **6-24** | 2087 | 1124 | 1.01  (0.88-1.15) | 1.04  (0.91-1.19) |
| Flunitrazepam | 18-26 ^b,^^ | 1 (0.0) | 1 (100) | - | - |
| Estazolam | 10-24 ^a,^^ | 13 (0.4) | 7 (53.9) | 1.00  (0.34-2.99) | 0.89  (0.29-2.79) |
| Bromazepam | 17 (10-20) ^b,#^ | 326 (9.0) | 169 (51.8) | 0.92  (0.73-1.15) | 0.92  (0.73-1.16) |
| Lorazepam | 14±5 ^a,°^ | 782 (21.5) | 452 (57.8) | **1.23**  **(1.05-1.44)** | **1.26**  **(1.07-1.49)** |
| Pinazepam | 10-15 ^b,^^ | 1 (0.0) | 1 (100) | - | - |
| Alprazolam | 12±2 ^a,°^ | 667 (18.3) | 351 (52.6) | 0.94  (0.80-1.12) | 0.94  (0.79-1.11) |
| Lormetazepam | 11 (10-12) ^b,#^ | 283 (7.8) | 135 (47.7) | **0.77**  **(0.60-0.98)** | 0.83  (0.65-1.06) |
| Clordiazepossido | 10±3.4 ^a,°^ | 4 (0.1) | 3 (75.0) | 2.58  (0.27-24.82) | 2.28  (0.23-22.10) |
| Oxazepam | 8.0±2.4 ^a,°^ | 10 (0.3) | 5 (50) | 0.86  (0.25-2.97) | 0.94  (0.27-3.28) |
| **Short-acting BZD** | **≤6** | 282 | 144 | 0.89  (0.70-1.13) | 0.90  (0.70-1.15) |
| Brotizolam | 4.4 (3.6-7.9) ^b,#^ | 50 (1.4) | 32 (64.0) | 1.54  (0.86-2.75) | 1.53  (0.85-2.74) |
| Clotiazepam | 4 (3-15) ^b,#^ | 8 (0.2) | 4 (50.0) | 0.86  (0.21-3.44) | 0.77  (0.19-3.12) |
| Etizolam | 3.4±0.3 ^b,°^ | 20 (0.6) | 8 (40.0) | 0.57  (0.23-1.40) | 0.54  (0.22-1.34) |
| Triazolam | 2.9±1.0 ^a,°^ | 177 (4.9) | 93 (52.5) | 0.95  (0.70-1.28) | 0.97  (0.71-1.32) |
| Clorazepato | 2.0±0.9 ^a,°^ | 3 (0.1) | 2 (66.7) | 1.72  (0.16-8.97) | 1.34  (0.12-14.86) |
| Midazolam | 1.9±0.6 ^a,°^ | 24 (0.7) | 5 (20.8) | **0.22**  **(0.08-0.60)** | **0.21**  **(0.07-0.64)** |
| **Z-drugs** | **≤6** | 358 | 179 | 0.84  (0.68-1.05) | 0.87  (0.70-1.08) |
| Zopiclone | 5 (3.8-6.5) ^b,#^ | 5 (0.1) | 3 (60) | 1.29  (0.22-7.72) | 1.17  (0.19-7.11) |
| Zolpidem | 2.4±0.2 ^b,°^ | 353 (9.7) | 176 (49.9) | 0.84  (0.67-1.05) | 0.86  (0.69-1.08) |

*^a^ Goodman and Gilman’s the Pharmacological Basis of Therapeutics, 15^th^ Edizione, ^b^ PubChem U.S. National Library of Medicine (PubChem).*

^#^ Median (range); ^°^ mean ± standard deviation; ­^^^range.

*Adjusted by age, sex, Caucasian ethnicity, presence of concomitant drugs and concomitant conditions.

**Supplementary table 3.** Risk of hospitalization associated with the different active substances belonging to opioids

| **Suspected**  **medications** | **Suspected adverse events** | **Suspected adverse events requiring hospitalizations** | **Crude ROR** | **Adjusted ROR*** |
| --- | --- | --- | --- | --- |
|  | **N=2884 (%)** | **N=727 (%)** |  |  |
| **Opioids** | | | | |
| **Strong opioids** | 574 | 197 | **1.75**  **(1.44-2.14)** | **1.53**  **(1.25-1.87)** |
| Morphine | 83 (2.9) | 33 (39.8) | **2.00**  **(1.28-3.14)** | **1.76**  **(1.11-2.79)** |
| Hydromorphone | 17 (0.6) | 7 (41.2) | 2.09  (0.79-5.50) | 1.64  (0.61-4.36) |
| Oxycodone | 84 (2.9) | 29 (34.5) | **1.59**  **(1.01-2.51)** | 1.44  (0.91-2.29) |
| Pethidine | 5 (0.2) | 2 (40.0) | 1.98  (0.33-11.88) | 1.67  (0.27-10.14) |
| Fentanyl | 169 (5.9) | 59 (34.9) | **1.64**  **(1.18-2.28)** | 1.40  (0.99-1.96) |
| Buprenorphine | 73 (2.5) | 26 (35.6) | **1.67**  **(1.02-2.71)** | 1.44  (0.88-2.36) |
| Tapentadol | 143 (5.0) | 41 (28.7) | 1.20  (0.83-1.75) | 1.13  (0.77-1.66) |
| **Weak opioids** | 711 | 153 | **0.76**  **(0.62-0.94)** | 0.83  (0.68-1.02) |
| Tramadol | 711 (24.7) | 153 (21.5) | **0.76**  **(0.62-0.94)** | 0.83  (0.68-1.02) |
| **Fixed Associations** | 1599 | 377 | **0.82**  **(0.70-0.98)** | 0.85  (0.72-1.01) |
| Oxycodone + Naloxone | 441 (15.3) | 117 (26.5) | 1.09  (0.86-1.37) | 0.98  (0.78-1.24) |
| Oxycodone + Paracetamol | 4 (0.1) | 0 (0) | - | - |
| Codeine + Paracetamol | 847 (29.3) | 205 (24.2) | 0.93  (0.77-1.12) | 0.99  (0.82-1.20) |
| Tramadol + Dexketoprofen | 113 (3.9) | 8 (7.1) | **0.22**  **(0.11-0.45)** | **0.26**  **(0.13-0.54)** |
| Tramadol + Paracetamol | 193 (6.7) | 46 (23.8) | 0.99  (0.66-1.30) | 0.93  (0.65-1.32) |
| Morphine + Antispasmodics | 1 (0.0) | 1 (100) | - | - |

*Adjusted by age, sex, Caucasian ethnicity, presence of concomitant drugs and concomitant conditions.
